# Predictive factors of thoracic aortic calcification in patients candidate for cardiac surgery

**DOI:** 10.1186/s13019-024-02636-8

**Published:** 2024-03-23

**Authors:** Amin Bagheri, Shapour Shirani, Arash Jalali, Shahrzad Salehbeigi, Jamshid Bagheri

**Affiliations:** 1 Cardiovascular Diseases Research Institute, Tehran Heart Center, Tehran University of Medical Sciences, Tehran, Iran; 2 Cardiovascular Research Center, Shahid Beheshti University of Medical Sciences, Tehran, Iran; 3 Department of Cardiac Surgery, Tehran Heart Center, Tehran University of Medical Sciences, Tehran, Iran

**Keywords:** Thoracic aortic calcification, Cardiac surgery, Thoracic CT, Predictors, Aortic disease

## Abstract

**Background:**

The presence of the severe thoracic aortic calcification (TAC) in cardiac surgery patients is associated with adverse post-operative outcome. However, the relationship between cardiovascular risk factors and aortic plaque burden remains unknown. The objective of this study was to determine the predictive factors of TAC in patients candidate for cardiac surgery.

**Methods:**

Patients who underwent thoracic CT scan prior to cardiac surgery between August 2020 to April 2021 were included. Of 556 patients, 209 (36.7%) had a thoracic aortic calcium score (TACS) ≥ 400 mm [3] and were compare with the remaining patients. Predictors of severe TAC were assessed through stepwise multivariable logistic regression analysis.

**Results:**

The patients with TACS ≥ 400 had a higher mean age (67.3 ± 7.1 vs. 55.7 ± 10.6; *p* < 0.001) with a higher frequency of diabetes mellitus (40.7% vs. 30.8%; *p* = 0.018), dyslipidemia (49.8% vs. 38.6%; *p* = 0.010), hypertension (60.8% vs. 44.7%; *p* < 0.001), opium addiction (18.2% vs. 11.2%; *p* = 0.023), peripheral vascular disease (PVD) (7.7% vs. 2.3%; *p* = 0.005) as compared with TACS < 400. The multiple determinants of TAC were PVD (OR = 2.86) followed by opium addiction, diabetes and age.

**Conclusions:**

Thoracic CT scan prior to cardiac surgery for patients with older age, diabetes, opium addiction and PVD is recommended. Our study could serve as a foundation for future research endeavors aimed at establishing a risk score for TAC.

## Introduction

Cardiovascular disease is one of the major causes of death. Thoracic aortic calcification (TAC) reflects systemic atherosclerosis and is associated with increased risk of cardiovascular events owing to its large surface which is particularly prone to vulnerable plaques. Detection and quantification of TAC can be achieved through thoracic computed tomography (CT) scans, employing methods such as the Agatston method [1–3]. Cardiac surgeries including coronary artery bypass grafting, valve and ascending aortic surgeries require cross-clamping of the aorta, which can dislodge the calcified atherosclerosis plaques and increase the risk of ischemic events such as cerebrovascular accident [1]. Moreover, severe TAC, patients may be labeled as having a porcelain aorta, posing challenges for safe cannulation and aortic cross-clamping. This condition represents the most common reason for technical inoperability [4, 5].

The extent of TAC, determined through preoperative CT scan, is independently associated with increased long term mortality in patients underwent cardiac surgery [6]. However, the routine use of thoracic CT scan prior to cardiothoracic surgery is not recommended due to associated radiation exposure and high costs. Notably, TAC may be incidentally identified during a coronary artery calcium (CAC) scan. It’s worth mentioning that conventional CT scans for CAC typically exclude common sources of calcification, such as the aortic arch and proximal descending aorta [7].

The objective of this study was to determine the relationships between cardiovascular risk factors and severe aortic calcification. In this context, we examined a consecutive series of patients eligible for cardiac surgery who underwent a thoracic CT scan, a routine preoperative imaging procedure implemented during the Covid-19 Pandemic. The aim was to identify the predictive factors associated with severe TAC.

## Materials and methods

### Study population

This retrospective single-center study was conducted between August 2020 to April 2021 at Tehran Heart Center (THC). The patient’s selection criteria were anyone who admitted to our center and candidate for cardiac surgery during COVID pandemic. Patients with cardiogenic shock or previous history of cardiac or thoracic surgery and those with thoracic aortic aneurysm were excluded. The main indication for surgery were coronary artery disease and valvular disease. Patients were divided into two groups based on thoracic aortic calcium score (TACS). All data were collected from THC databases. There is a complete dataset with no missing information as all patients were admitted to our center and candidate for cardiac surgery and we retrospectively assessed the imaging (CT scan) data. This study was approved by the ethics committee of THC and all patients provided informed consent.

The patient characteristics including age, gender, hypertension, diabetes mellitus, dyslipidemia, opium addiction, smoker, recent myocardial infarction, left ventricular ejection fraction (LVEF) and renal failure in addition to peripheral vascular disease (PVD) and previous cerebrovascular accident (CVA) were gathered. Hypertension was defined as having at least one of the following conditions: a minimum systolic blood pressure of 140mmHg, a minimum diastolic blood pressure of 90mmHg, or a history of taking antihypertensive drugs. Dyslipidemia was defined as having either a minimum total cholesterol of 240 mg/dL, a minimum triglyceride of 200 mg/dL, a minimum low-density lipoprotein cholesterol level of 160 mg/dL, low levels of high-density lipoprotein cholesterol (< 40 mg/dL in men and < 50 mg/dL in women), or a history of taking lipid-lowering drugs. Cigarette smoking and opium consumption were determined based on the patient’s self-reported status. A current smoker was defined as someone who currently smokes and has smoked more than 100 cigarettes in the past. Opium addiction was defined as inhaling opium smoke and/or eating opium in its crude form at least three times a week. Diabetes mellitus was defined according to the American Diabetes Association as having a definite history of diabetes with records of treatment, fasting blood sugar levels ≥ 126 mg/dL, or 2-hour postprandial glucose levels ≥ 200 mg/dL. A recent MI was characterized as a documented MI occurring within the past 21 days. Elevated serum creatinine level more than 120 mmol/L made a category of renal failure. PVD was defined by an ankle-brachial index ≤ 0.9.

### Thoracic aortic calcification

TAC was defined as calcification of the ascending, arch and descending segments of thoracic aorta thereby using Non-enhanced multi slice CT scans. All images were reconstructed with a thickness of 2.5 mm. A CT threshold of 130 Hounsfield units and 4 pixels was used to identify a calcified lesion and quantified using the Agatston method. Total calcification of thoracic aorta was assessed by summing weighted TACSs of all calcified lesions by an experienced radiologist who was blinded to the patient’s medical records. Based on the categories of TACS, defined in the previous studies, TACS ≥ 400 was considered as severe aortic calcification [3, 8].

### Statistical analysis

Categorical data are presented as frequency with percentage, and continuous data are described as mean with standard deviation (SD) for normally distributed data and median with 25th and 75th percentiles for non-normally distributed data. Categorical variables were compared between TAC groups by Chi-square or Fisher’s exact test, as appropriate. Student t-test and Mann-Whitney U test were used for comparison of normally and non-normally distributed data between two groups, respectively. Logistic regression models were used to evaluate the effect of covariates on severe TAC. All variables which their univariate effects have *p*-values less than 0.1 were candidate to enter the multivariable model. a multivariable logistic regression model with backward elimination method was used to detect the best determinants of the severe TC. Effects were expressed as odds ratio (OR) and 95% confidence interval (CI). Calibration belt was used to assess the calibration of the final multivariable model and the area under the receiver operating characteristic (ROC) curve was used to evaluate the discrimination power of the final model. All statistical analyses were conducted using Stata statistical software, release 14.2 (College Station, TX: Stata Corp.).

## Results

### Patient characteristics

Demographic data and characteristics of the patients are shown in Table 1. Of 556 patients, 209 (36.7%) have TACS ≥ 400 mm^3^ and they were compare with the rest of patients. The patients with TACS ≥ 400 had a higher mean age (67.3 ± 7.1 vs. 55.7 ± 10.6; *p* < 0.001) with a higher frequency of diabetes mellitus (40.7% vs. 30.8%; *p* = 0.018), hypertension (60.8% vs. 44.7%; *p* < 0.001), dyslipidemia (49.8% vs. 38.6%; *p* = 0.010), opium addiction (18.2% vs. 11.2%; *p* = 0.023), peripheral vascular disease (PVD) (7.7% vs. 2.3%; *p* = 0.005) as compared with TACS < 400.

**Table 1:** Patient Characteristics and predictors of severe thoracic aortic calcification

Predictor	TACS < 400 (*n* = 347)	TACS ≥ 400 (*n* = 209)	Univariate		Multivariable	
			OR (95% CI)	*P* value	OR (95% CI)	*P* value
Mean age, y	55.7 ± 10.6	67.3 ± 7.1	1.17 (1.14–1.20)	< 0.001	1.17 (1.14–1.21)	< 0.001
Female	131 (37.8)	84 (40.2)	0.90 (0.63–1.28)	0.567	-	-
BMI, kg/m^2^	27.5 ± 4.4	27.9 ± 4.3	1.02 (0.98–1.06)	0.298	-	-
Diabetes mellitus	107 (30.8)	85 (40.7)	1.54 (1.07–2.20)	0.018	1.70 (1.09–2.65)	0.019
Hypertension	155 (44.7)	127 (60.8)	1.92 (1.35–2.72)	< 0.001	1.31 (0.79–2.17)	0.300
Dyslipidemia	134 (38.6)	104 (49.8)	1.57 (1.11–2.23)	0.010	1.27 (0.77–2.21)	0.347
Family history of CAD	103 (29.8)	65 (31.4)	1.08 (0.74–1.57)	0.686	-	-
Opium Addiction	39 (11.2)	38(18.2)	1.75 (1.08–2.85)	0.023	1.87 (1.04–3.35)	0.037
Current Smoking	78 (22.5)	55 (26.3)	1.23 (0.83–1.83)	0.305	-	-
Recent MI	24 (6.9)	9 (4.3)	0.61 (0.28–1.33)	0.211	-	-
LVEF, %	47.4 ± 9.2	45.9 ± 9.5	0.98 (0.96-1.00)	0.069	0.98 (0.96–1.01)	0.124
PVD	8 (2.3)	16 (7.7)	3.51 (1.48–8.36)	0.005	2.86 (0.90–9.10)	0.075
Previous CVA	19 (5.5)	18 (8.6)	1.63 (0.83–3.18)	0.154	-	-
Renal Failure	36 (10.4)	28 (13.4)	1.33 (0.79–2.26)	0.286	-	-
Valve surgery Indication	33 (9.5)	29 (13.9)	1.53 (0.90–2.61)	0.115	-	-

Data are presented frequency (percentage), or mean ± standard deviation

The area under the ROC curve for aortic calcification was 83.5% (95% CI: 80.2–86.8%, *P* value < 0.001)

TACS, thoracic aortic calcium score; OR, odds ratio; CI, confidence interval; BMI, body mass index; CAD, coronary artery disease; MI, myocardial infarction; LVEF, left ventricular ejection fraction; PVD, peripheral vascular disease; CVA, cerebrovascular accident; ROC, receiver operating characteristic

### Predictors of severe thoracic aortic calcification

**Fig. 1:**
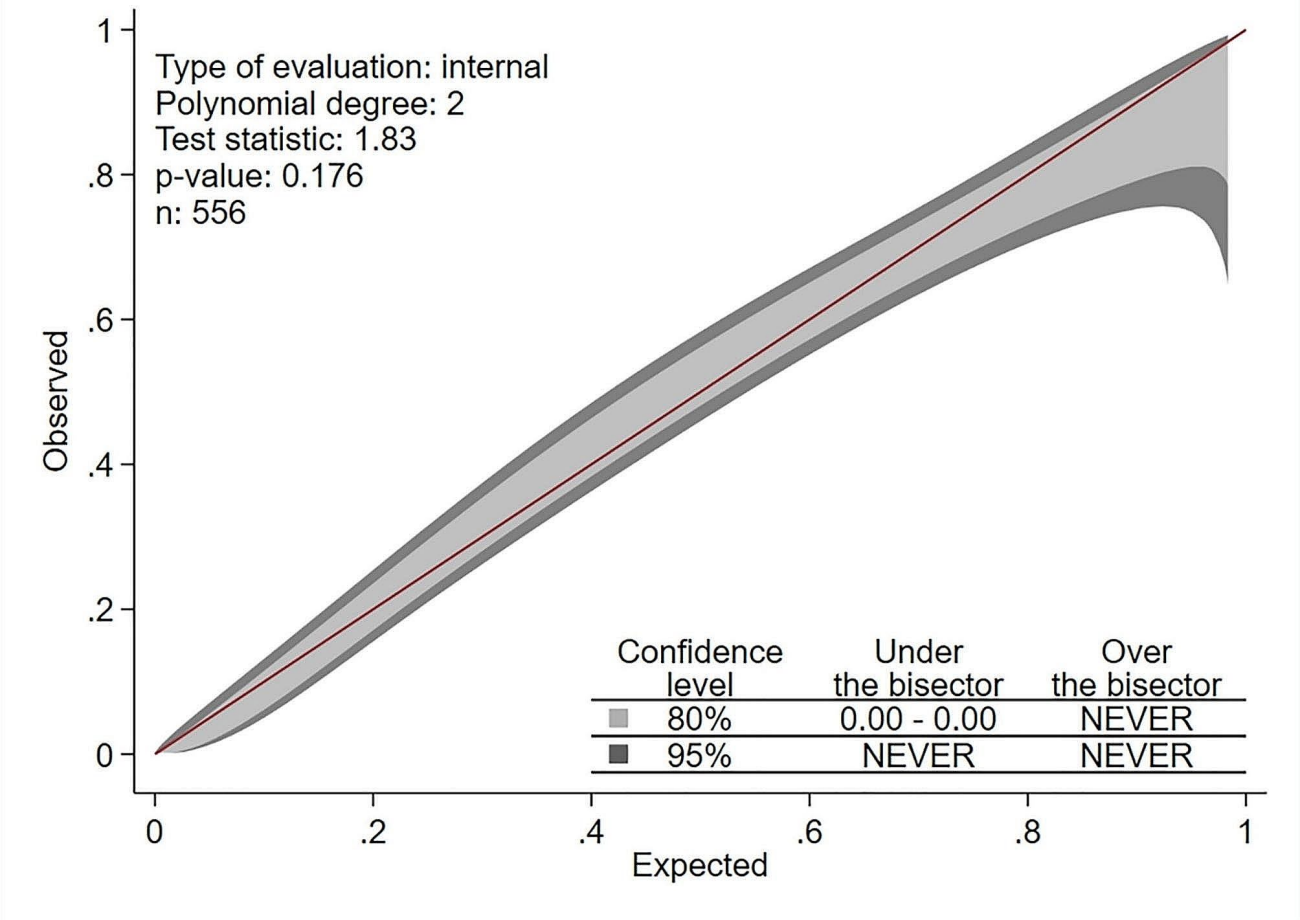
The calibration belt for predictors of severe TAC showed adequate calibration of the model using polynomial logistic regression

Multivariable logistic regression analyses revealed that age (OR = 1.17 [1.14–1.21]; *p* < 0.001), diabetes mellitus (OR = 1.70 [1.09–2.65]; *p* = 0.019), opium addiction (OR = 1.87 [1.04–3.35]; *p* = 0.037) and PVD (OR = 2.86 [0.90–9.10]; *p* = 0.075) are significantly associated with severe TAC (Table 1). The calibration belt for predictors of severe TAC revealed adequate calibration of the model using polynomial logistic regression (*p* = 0.176) (Fig. 1). The area under the ROC curve for thoracic aortic calcification was 83.5% (95% CI: 80.2–86.8%, *p* < 0.001).

## Discussion

In the present study, we evaluated the characteristics of patients who were candidate for cardiac surgery and determined their relationship with the severity of TAC on chest CT scan. Our finding indicated that age, diabetes, opium addiction and PVD are significantly associated with severe TAC.

Calcification of aorta has been shown to be associated with increased risk of Cardiovascular morbidity and mortality [7]. The location, characteristics and associated risks of TAC have been described with echocardiography for decades [9]. However, severe calcification can confound the echocardiographic assessment owing to acoustic shadowing and reverberation artifact. On the other hand, CT scan has improved spatial resolution. Furthermore, ultrasound physics are not easily amenable to quantify calcification unlike the Agatston method for CT [3, 10]. Nonetheless, pre-operative thoracic CT is not recommended routinely. Recently, Nates et al. discovered that a routine preoperative thoracic CT scan in all non-urgent cardiac surgical patients effectively reveals both cardiac and extra-cardiac findings. Emphasizing the necessity of conducting preoperative thoracic CT scan for all individuals undergoing cardiac surgery is important. However, it is essential to recognize that this practice is associated with increased costs, radiation exposure, and potential risks, particularly for younger women of reproductive age. Consequently, future research should identify specific demographics that would derive the most benefit from preoperative screening. Furthermore, additional studies are warranted to assess the long-term impact of these screenings on morbidity and mortality [11].

During COVID-19 pandemic, suspected patients who were candidate for cardiac surgery underwent thoracic CT scan. The main purpose of this study was to determine the high risk patients for severe TAC. Based on previous studies, TAC > 400 increased the risk of ischemic or hemorrhagic stroke significantly. Hermann et al. found that TAC ≥ 400 category carried a 9.21-fold stroke risk [1]. Furthermore, another study demonstrated that the rate of coronary artery calcification increased up to 65.4% after 5 years in participants with TAC ≥ 400 at baseline [12]. Consequently, patients were categorized into two groups according to their TAC scores.

Aortic calcium in either the ascending or descending thoracic aorta has been shown to be related closely to coronary artery calcification, suggesting a common underlying systemic vascular atherosclerotic process [13, 14]. Thoracic aortic atheroma burden is extended. Kurra et al. revealed that more than 70% cardiac surgical patients had plaque in both the ascending and descending part of the aorta. They found that There was a strong correlation between the ascending and descending total plaque [6]. Therefore, we decided to assess all calcified lesions in the ascending, arch and descending segments of thoracic aorta.

Pathophysiologic processes of arterial calcification include calcification of the intimal layer, as part of atherosclerotic plaque development, and calcification of the media which is prominent in diabetes mellitus and other metabolic disorders [15]. Moreover, hyperglycemia has been reported to promote calcification by increasing expression of bone morphogenic proteins 2 and 4 and procalcific molecules such as osteopontin [16, 17]. Consistent with previous studies [18–20], older age and diabetes as determinants was significantly associated with TAC.

While patients with calcification were more likely male, we did not find gender to be significantly associated with TAC (Table 1) and there was no gender interaction noted in the relation of low ankle-brachial index. This interestingly differs from several prior studies that report a greater prevalence amongst women of calcium in the thoracic aorta [21, 22].

Opioid derivations have been shown to have an immunomodulatory effect. Experimental studies revealed that opioids could accelerate the formation of atherosclerotic plaques in vessels by raising the monocyte chemotactic protein-1 level and apoptosis frequency [23–25]. Recently, opium addiction has been shown to have significant association with the formation of internal carotid artery plaques [26]. Our study revealed that Opium addiction significantly increased the severity of TAC (about 87%). Moreover, current smoking and hypertension had higher prevalence in participants with calcification of thoracic aorta. However, regression analysis showed the incident of TAC was not significantly associated with either current cigarette smoking or hypertension unlike previous studies [12, 18–20]. In MESA (Multi-Ethnic Study of Atherosclerosis) trial, traditional cardiovascular risk factors were associated with aortic calcification. Although current smoking and hypertension had strongest associations [18]. Our findings indicated that TAC was most strongly associated with PVD (OR = 2.86) as defined by an ankle-brachial index (ABI) ≤ 0.9. Previous observational study among patients with diabetic showed the association of TAC with low ABI [27]. Although either high (≥ 1.4) or low ankle-brachial indexes confer increased cardiovascular risk and are considered abnormal, we determined to focus on low ABI in this study due to greater evidence of cardiovascular risk related to low ABI [28–30].

The retrospective nature of the present study is one of its limitations. Furthermore, our study was conducted at a tertiary care referral center that could lead to referral bias. In addition, the small number of patients in our study and parameters included in the analysis may have affected study power. In this study, the thoracic aorta was examined in the available location on initially performed thoracic CT scan, excluding the infrarenal abdominal aorta, a location with noted higher prevalence of calcification [18–20]. Consequently, our TAC scores did not evaluate the potential importance of abdominal aorta calcification to predict TAC incidence or progression.

In conclusion, we recommended to perform thoracic CT prior to cardiac surgery for patients with older age, diabetes, opium addiction and PVD, not as a routine. Our study could serve as a foundation for future research endeavors aimed at establishing a risk score, incorporating a more extensive sample size. Additionally, exploring outcomes through further analysis could be identified as another potential avenue for future investigation.

## Data Availability

The data that support the findings of this study are available on request from the corresponding author (J.B).
